# Increase in SARS-CoV-2 Seroprevalence in UK Domestic Felids Despite Weak Immunogenicity of Post-Omicron Variants

**DOI:** 10.3390/v15081661

**Published:** 2023-07-30

**Authors:** Grace B. Tyson, Sarah Jones, Chloe Montreuil-Spencer, Nicola Logan, Sam Scott, Hagar Sasvari, Michael McDonald, Leigh Marshall, Pablo R. Murcia, Brian J. Willett, William Weir, Margaret J. Hosie

**Affiliations:** 1 MRC-University of Glasgow Centre for Virus Research, Glasgow G61 1QH, UK; 2 School of Biodiversity, One Health and Veterinary Medicine, University of Glasgow, Glasgow G61 1QH, UK

**Keywords:** serology, SARS-CoV-2, coronaviruses, cats, feline infections, neutralising antibodies, ELISA

## Abstract

Throughout the COVID-19 pandemic, SARS-CoV-2 infections in domestic cats have caused concern for both animal health and the potential for inter-species transmission. Cats are known to be susceptible to the Omicron variant and its descendants, however, the feline immune response to these variants is not well defined. We aimed to estimate the current seroprevalence of SARS-CoV-2 in UK pet cats, as well as characterise the neutralising antibody response to the Omicron (BA.1) variant. A neutralising seroprevalence of 4.4% and an overall seroprevalence of 13.9% was observed. Both purebred and male cats were found to have the highest levels of seroprevalence, as well as cats aged between two and five years. The Omicron variant was found to have a lower immunogenicity in cats than the B.1, Alpha and Delta variants, which reflects previous reports of immune and vaccine evasion in humans. These results further underline the importance of surveillance of SARS-CoV-2 infections in UK cats as the virus continues to evolve.

## 1. Introduction

Felids have been established as one of the most commonly infected non-human hosts of SARS-CoV-2. Domestic cats, in particular, are known to be highly susceptible to SARS-CoV-2 infections [1,2,3,4], most of which are believed to result from separate anthropozoonotic transmission events, in which the virus is transmitted from owner to pet, rather than cat-to-cat [5]. Despite this, cat-to-cat transmission has been demonstrated under experimental conditions [6,7,8] and SARS-CoV-2 antibodies have been detected in both abandoned and stray cats [9,10], which may indicate other possible routes of transmission. Transmission has also been detected between cats and other animals such as mink [11].

In the UK, moderate levels of SARS-CoV-2 seroprevalence in domestic cats have been documented in multiple publications [12,13,14], based on the analysis of remnant diagnostic samples submitted for other purposes. Despite this appreciable seroprevalence, veterinary diagnostic testing of domestic animals for SARS-CoV-2 is rarely performed due to the very narrow testing criteria dictated by government guidelines. Consequently, it is difficult for veterinarians to confirm suspected cases of the disease, which can lead to improper treatment of the infection. As a result, there exists a dearth of knowledge regarding the long-term effects and clinical pathologies associated with SARS-CoV-2 infection in cats, with clinicians and owners potentially unaware of the cause of a pet’s illness. The lack of diagnostic testing means that the true scale of animal SARS-CoV-2 cases cannot be fully appreciated and that the transmission dynamics associated with feline infections remain poorly understood. Despite the majority of feline cases appearing to be owner-to-cat transmission events [15,16,17], felid-to-human transmission has been documented from a cat [18] and a lion [19]. It is currently unknown whether felids can infect other susceptible animals, such as dogs; however, this remains a possibility as cats and dogs frequently live together in the same household [20,21,22].

Currently, the majority of reported natural SARS-CoV-2 infections in felids have been either asymptomatic or subclinical [23], however, severe sequelae including acute respiratory distress [24] and myocarditis [25,26] have been documented.

Cats are known to generate a robust neutralising antibody response to SARS-CoV-2, which enables animals to resist re-infection [27], as well as an IgG response against the Spike and Nucleocapsid proteins of multiple different variants [14,21,28].

There is a distinct lack of knowledge, however, of the feline immune response to more recent, post-Omicron variants. In the UK, there have been multiple novel predominant circulating variants since the peak of the Omicron (BA.1) wave in December 2021–January 2022 [29]. These are: BA.2 (descended directly from BA.1) [30], BA.5 (descended from BA.2) [31], BQ.1.1 (descended from BA.5) [32] and XBB (a recombinant of two different BA.2 strains) [33], all of which evolved from the Omicron (BA.1) variant. In humans, all four of these newer variants [34,35], as well as Omicron (BA.1) [36], have been shown to evade vaccine- and infection-derived immunity, as well as reduce the efficacy of monoclonal antibody therapy [37].

We previously observed that distinct patterns of immunity to different variants arise depending on which variant was likely to have infected the animal. Furthermore, this serological study demonstrated that the emergence of novel variants in the feline population mirrored and trailed their appearance in the human population [14]. The aims of this study were to characterise the neutralising antibody response to the Omicron (BA.1) variant and its descendants, to provide an updated picture of seroprevalence and to investigate the prevalence of non-virus neutralising anti-SARS-CoV-2 IgG in UK cats.

## 2. Materials and Methods

### 2.1. Samples

Residual serum and plasma samples for serological testing were provided by the University of Glasgow Veterinary Diagnostic Services laboratory (VDS). These samples had been submitted by practising UK veterinary clinicians for diagnostic testing including routine monitoring, pre-breeding screening, detection of infections and other clinical pathology tests. Only residual samples that remained after testing were used for this study and none of the samples had been submitted to the VDS because of suspected SARS-CoV-2 infection. Ethical approval for the study was granted by the University of Glasgow Veterinary Ethics Committee (EA27/20). The investigators (GT, NL, CMS, SS, HS and SJ) were blinded to sample metadata until the data analysis stage as samples were labelled with a unique 6-digit identifier.

### 2.2. Serological Testing

Serum and plasma samples were screened for SARS-CoV-2 neutralising antibodies at a fixed dilution of 1 in 50 using a pseudotype-based virus neutralisation assay (PVNA). Subsequently SARS-CoV-2 neutralising antibody titres against each variant were calculated for all samples that tested positive in the screening assay, by performing a PVNA with serially diluted samples.

Samples collected early in the pandemic were initially tested against pseudotype virus bearing the S protein of the ancestral variant (Wuhan-Hu-1 D614G (B.1)) only and new variants were subsequently included in the assay over time, as they emerged in the UK population.

### 2.3. Pseudotype-Based Virus Neutralisation Assay

The method for this assay has been described previously [14,38]. Briefly, HEK293T and HEK293-ACE2 cells (described previously [39]) were maintained in Dulbecco’s modified Eagle’s medium (DMEM) supplemented with 10% foetal bovine serum, 200 mM L-glutamine, 100 µg/mL streptomycin and 100 IU/mL penicillin. HEK293T cells were transfected with the appropriate SARS-CoV-2 S gene expression vector (wild type or variant) in conjunction with p8.91 [40] and pCSFLW [41] using polyethylenimine (PEI, Polysciences, Warrington, PA, USA). HIV (SARS-CoV-2) pseudotypes were harvested from culture fluids 48 h post-transfection, filtered at 0.45 µm, aliquoted and frozen at −80 °C prior to use. The SARS-CoV-2 spike glycoprotein expression constructs were synthesised by GenScript (Rijswijk, The Netherlands). Construct mutations relative to the Wuhan-Hu-1 sequence are detailed in Section 1 of the Supplementary Information.

All synthesised S genes were codon-optimised, incorporated the mutation K1255STOP to enhance surface expression and were cloned into the pcDNA3.1(+) eukaryotic expression vector. 293-ACE2 target cells [41] were maintained in complete DMEM supplemented with 2 µg/mL puromycin.

The fixed dilution screen was performed with serum/plasma diluted 1:50 in complete DMEM (in duplicate) for each pseudotype. Diluted samples were incubated with HIV (SARS-CoV-2) pseudotypes for 1 h and plated onto 239-ACE2 target cells. After 48–72 h, luciferase activity was quantified by the addition of Steadylite Plus chemiluminescence substrate and analysis on a Perkin Elmer EnSight multimode plate reader (PerkinElmer, Beaconsfield, UK). Samples which reduced the infectivity of the pseudotypes by at least 90% were classed as positive. For positive samples, SARS-CoV-2 neutralising activity was then quantified by serial dilution. Each sample was serially diluted (in triplicate) from 1:50 to 1:36,450 in complete DMEM prior to incubation with the respective viral pseudotype. Antibody titre was then estimated by interpolating the dilution of serum, which reduces the mean infectivity of the triplicate sample to 10% of the value for the no serum pseudotype control.

For the detection of SARS-CoV-2 antibodies, live virus neutralisation assays have been shown to have a high level of correlation with pseudovirus-based assays, with both VSV and lentiviral backbones [42,43].

### 2.4. Feline-Specific Secondary ELISA

Following supply issues with the commercial DABA kit initially used in this study (Microimmune SARS-CoV-2 Double Antigen Bridging Assay (COVT016), Clin-Tech, Guildford, England), an in-house feline-specific ELISA was designed by combining and adapting protocols established previously by Parr et al. (FeLV) [44] and Hughes et al. (Human SARS-CoV-2) [39].

ELISA plates (Immulon 2HB Flat Bottom MicroTiter Plates, ImmunoChemistry Technologies, Davis, CA, USA) were coated with SARS-CoV-2 RBD protein diluted to 50 ng per well in 100 mM sodium bicarbonate and 33 mM anhydrous sodium carbonate buffer for 1 h at room temperature, washed with 1x PBS with 0.1%Tween-20 (wash buffer) and then blocked for a further hour with a 1x solution of casein (Vector Laboratories, Newark, CA, USA) diluted in wash buffer.

Plates were then washed before samples diluted to 1:1000 in casein buffer were added to wells in duplicate and then incubated at room temperature for one hour. Three controls were used:

- No serum control—casein buffer.
- Negative control—pre-pandemic VDS cat samples that had previously tested negative for SARS-CoV-2 neutralising antibodies.
- Positive control—a pool of 20 pseudotype neutralisation assay and DABA positive serum samples identified previously [14].

Plates were washed and biotinylated anti-cat IgG (Vector Laboratories, Newark, CA, USA), diluted 1:8000 in casein buffer, was added to the plate and incubated for one hour at room temperature. Plates were washed again and streptavidin horseradish peroxidase (Vector Laboratories, Newark, CA, USA), diluted 1:8000 in casein buffer, was added and incubated for 20 min.

Plates were washed and 10x TMB super-slow substrate (Sigma-Aldrich, Burlington, MA, USA) was added and incubated at RT for 15 min. Dilute sulphuric acid was then added as a stop solution.

Within 10 min, the plates were read on a Multiskan plate reader (ThermoFisher, Waltham, MA, USA) at 450 nm to calculate optical density and at 620 nm to measure background transmission. Mean OD values for each sample were calculated from the duplicate measurements. A standard checkerboard assay was conducted to validate the assay against known positive and negative serum samples and to determine optimal concentrations of reagents for reducing background readings. With reference to the results of two other tests, two different ELISA cut-offs were defined (detailed in Section 2 of Supplementary Information), one with high stringency (OD ≥ 0.5, i.e., clearly positive) and one with low stringency (OD ≥ 0.1, i.e., equivocal).

### 2.5. Data Analysis

Duplicate samples were removed and samples from the same animal tested multiple times were identified; only the earliest positive sample was used to estimate seroprevalence.

Samples were categorised as either:

- Positive neutralising: positive (OD ≥ 0.5) or borderline (0.5 > OD ≥ 0.1) ELISA result plus a neutralisation titre ≥ 50 (dilution which results in a 90% reduction in infectivity) against at least one variant.
- Positive non-neutralising: positive ELISA result but no detectable neutralisation titre (titre of <50).
- Negative: negative ELISA result and no detectable neutralisation titre (titre < 50).

Sampling dates were categorised into seasons: winter (December to January), spring (March to May), summer (June to August) and autumn (September to November). The sampled cats were categorised into three breed type groups: purebred (e.g., Siamese and Ragdoll), non-purebred (e.g., domestic short hair) or unknown (no breed information available). Four age groups were defined: kitten/junior (<2 years), young adult (≥2 and <5 years), mature adult (≥5 and <10 years) and senior (≥10 years).

Data were analysed and graphs were prepared using GraphPad Prism 9.3.1. (GraphPad Software Inc, San Diego, CA, USA) and Microsoft Excel. Distribution of data was assessed using a Shapiro–Wilk normality test. Sample metadata (cat age, sex, breed, and postcode area), which had been supplied by submitting veterinarians, was acquired from the VDS database. Differences between groups were assessed for significance in paired data using a Wilcoxon test and in unpaired data using a Mann–Whitney test. Significance of categorical data was assessed using a chi-square test or Fisher’s Exact test when assumptions for each test were met. Correlations were assessed using Spearman’s test.

## 3. Results

### 3.1. Samples

After data cleaning, our dataset contained samples from 4385 cats.

**Figure 1:**
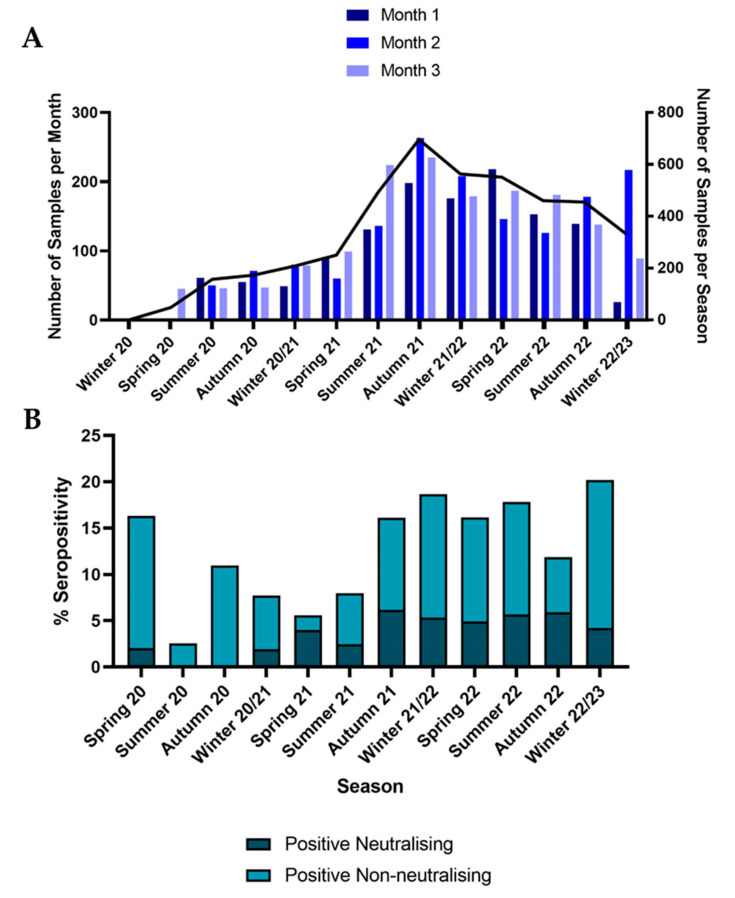
(**A**) Bar plot showing the number of samples included in the dataset per month on the left y axis. On the right y axis, the total number of samples per season in the dataset is shown as a black line. (**B**) Stacked bar plot showing the total percentage seropositivity across different seasons. The darker bars depict positive neutralising samples, and the paler stacked bars show positive non-neutralising samples.

Sampling dates ranged from January 2020 to February 2023 (Figure 1A).

Of the cats sampled in the study, 53% were male, 39% were female and 8% were of unknown sex. Their mean age was five years, the median was three years and when categorised by breed, 33% were purebred, 58% were non-purebred cats and 9% were of unknown breed.

The majority of the cats sampled in this dataset were located in England (76%), while 18% were from postcode areas within Scotland, 3% were from Wales, 2% from Northern Ireland and 1% from the Channel Islands and the Isle of Man. Sample numbers varied greatly by postcode area and this was not proportionate to the local human population. Over-represented areas included Glasgow, Edinburgh and Cambridge.

Samples which displayed a detectable neutralisation titre against at least one variant were categorised by the variant against which they produced the highest titre, and this is referred to as variant dominance. For example, a sample with its highest titre against the Alpha pseudotype is referred to as “Alpha dominant”.

### 3.2. Seroprevalence

Positive neutralising samples made up 4.4% of the collection (194/4385) (95%CI: 3.8%–5%). All positive samples, both positive neutralising and positive non-neutralising samples, comprised 13.9% of the dataset (609/4385) (95%CI: 12.9%–15%).

SARS-CoV-2 neutralising seroprevalence peaked in the autumn of 2021 with a high of 6.2% (95%CI: 4.4%–8%). Overall seropositivity peaked in the winter of 2022/23 at 20.2% (95%CI: 15.9%–24.5%) (Figure 1B). Neutralising seroprevalence has remained relatively stable since the autumn of 2021, between 4% and 6%. This is likely due to a combination of recent infections and a long-standing immunological response to older variants. There was a markedly lower ELISA positivity in proportion to neutralising positivity in spring/summer 2021; this time period corresponds to the peak of the Delta wave in humans. ELISA OD in positive neutralising samples was found to be significantly higher than in positive non-neutralising samples (*p* = 0.04). A higher ELISA OD also correlated with a higher neutralisation titre in positive neutralising samples (r = 0.47 (95%CI: 0.34–0.58), *p* < 0.0001).

#### 3.2.1. Comparison to Variants in Humans

**Figure 2:**
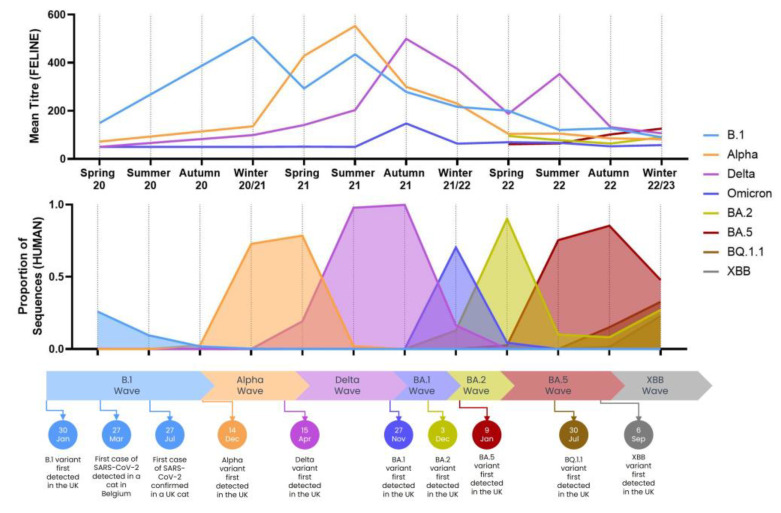
The upper chart shows the mean titre observed in positive neutralising feline samples and is plotted per season. The lower chart shows the proportion of human SARS-CoV-2 sequences identified as different variants over time; human data were obtained via GISAID CoV-Spectrum (GISAID). A timeline showing major events and emergences of new variants in the UK human population is shown at the bottom of this figure.

The emergence of variants in cats mirrored and trailed their appearance in humans when considering mean titres per season (Figure 2). As BA.1 and its descendent variants appear to be less immunogenic in cats, current peaks in feline immunity are no longer as defined as human waves. This is compounded by the fact that dominant titres against extinct variants, such as Alpha, are still being detected in 2023.

**Figure 3:**
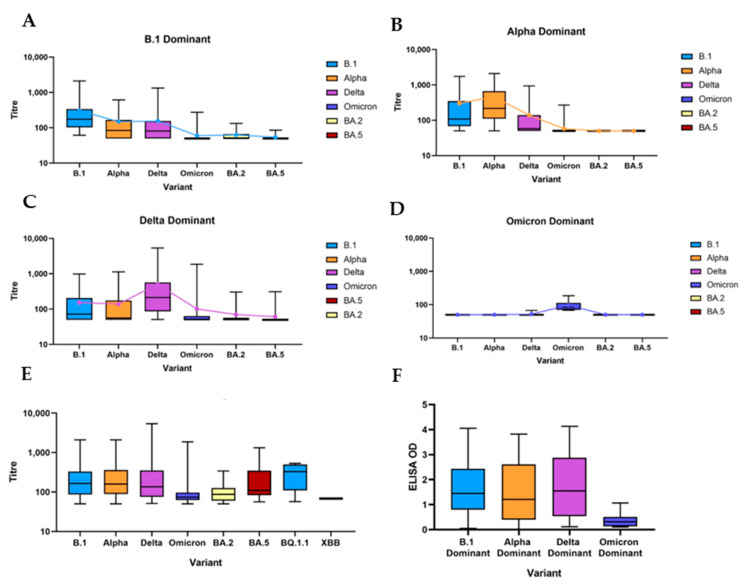
(**A**) Boxplot of B.1 dominant samples showing median neutralisation titres against different variants. The blue line shows the mean titre against each variant. (**B**) Boxplot of Alpha dominant samples showing median neutralisation titres against different variants. The orange line shows the mean titre against each variant. (**C**) Boxplot of Delta dominant samples showing median neutralisation titres against different variants. The purple line shows the mean titre against each variant. (**D**) Boxplot of Omicron dominant samples showing median neutralisation titres against different variants. The blue line shows the mean titre against each variant. (**E**) Boxplot showing median neutralisation titres against different variants from every neutralising positive sample. (**F**) Boxplot showing the median ELISA OD values of each group of variant dominant samples.

The mean overall titre against each variant, regardless of sample date or dominant variant, was calculated and is illustrated in Figure 3E. This provides a picture of both immunogenicity and levels of cross-neutralisation of different variants. The mean titres against Omicron and BA.2 were significantly lower than the titres against other variants (*p* < 0.0001 on the Kruskal–Wallis test).

#### 3.2.2. B.1 Dominant

Approximately one quarter (26% (50/194)) of all positive neutralising samples were B.1 dominant. On average, B.1 dominant samples showed moderate neutralisation of Alpha and Delta variants but low neutralisation of other variants (Figure 3A). Among B.1 dominant samples, the mean neutralising titre against B.1 was 306.

#### 3.2.3. Alpha Dominant

Alpha dominant samples comprised 23% (45/194) of positive neutralising samples. Alpha dominant samples showed moderate neutralisation both of B.1 and Delta, but very low neutralisation of all other variants tested; only 4 of 45 Alpha dominant samples had a detectable titre against Omicron (BA.1) (Figure 3B). The mean Alpha titre among Alpha dominant samples was 459.

#### 3.2.4. Delta Dominant

Delta dominant samples comprised 43% (84/194) of all positive neutralising samples. Delta dominant samples demonstrated moderate neutralisation of B.1 and Alpha and low neutralisation of both Omicron and BA.2 (Figure 3C). The mean titre of Delta dominant samples was 505.

#### 3.2.5. Omicron Dominant and Post-Omicron Dominant

It was found that 4% (8/194) of positive neutralising samples were Omicron dominant. Omicron dominant samples not only displayed a significantly lower mean dominant titre (93, *p* < 0.0001) compared to other variant dominant groups, but they also showed very low levels of neutralisation of other variants; just a single Omicron dominant sample displayed a detectable Delta titre, and none had detectable titres against any other variant (Figure 3D). Omicron dominant samples also had a significantly lower mean ELISA OD (*p* = 0.0037) than any other variant dominant sample (Figure 3F), strengthening the evidence for Omicron having lower immunogenicity in cats. The mean titre of Omicron dominant samples was 98, significantly lower than the mean dominant titre for B.1, Alpha and Delta dominant samples (*p* = 0.02).

Of the post-Omicron variants, one sample was found to be BA.2 dominant, four were BA.5 dominant and two were BQ.1.1 dominant. No samples were found to be XBB dominant. Due to low sample sizes, mean titres were not calculated for these dominance groups, however, all seven post-Omicron dominant samples showed negligible neutralisation of the pre-Omicron variants B.1, Alpha and Delta. However, cross-neutralisation of other post-Omicron variants was detected (Table 1).

### 3.3. Demographic Analysis

**Figure 4:**
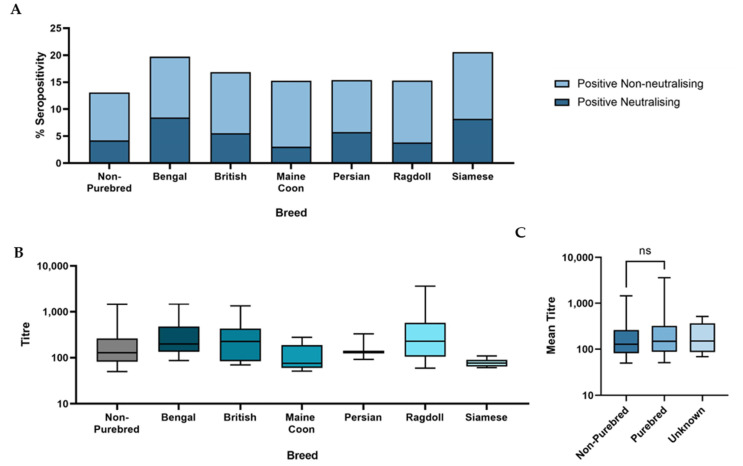
(**A**) Graph showing the total seroprevalence in different breeds of cat. The darker bars show neutralising seroprevalence and the stacked paler bars show non-neutralising seroprevalence. (**B**) Boxplot showing the mean titres of neutralising positive cats of different breeds. (**C**) Boxplot showing the mean titres of the three breed groups. There was no significant difference between the mean titres of purebred and non-purebred cats on a Mann–Whitney test (*p* = 0.2).

The strength of the antibody response in the different breed groups was similar and no significant difference in mean neutralisation titre was observed (*p* = 0.3) (Figure 4C). There was, however, a significant difference observed in SARS-CoV-2 neutralising seroprevalence, with purebred cats found to have significantly higher seroprevalence values than non-purebred cats (*p* = 0.008).

The breed groups were then sub-divided into specific breeds and those breeds with a sample size of *n* ≥ 50 were further analysed. A significant difference was observed between the mean neutralisation titres elicited by specific breed groups (*p* = 0.01) (Figure 4B), with Ragdoll and “British” breeds having the highest mean titres and Maine Coon and Siamese having the lowest. Although both Bengal and Siamese cats were more likely to be seropositive than other breeds, with both having a SARS-CoV-2 neutralising seroprevalence >8% (Figure 4A), this difference was not statistically significant.

**Figure 5:**
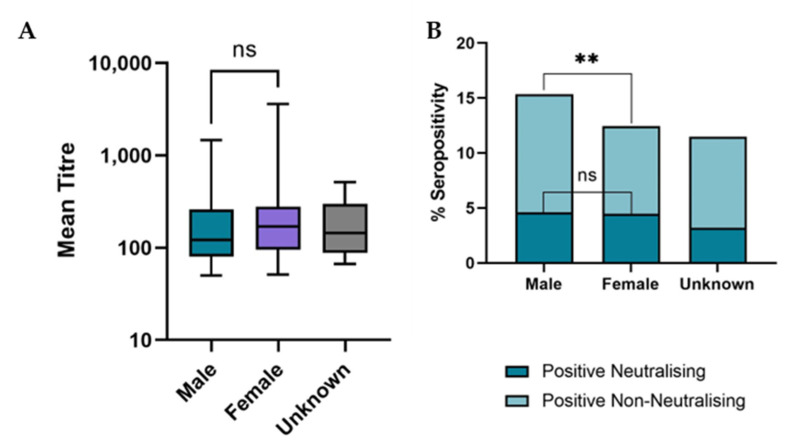
(**A**) Boxplot showing the mean titres of positive neutralising samples of each sex. It was observed that there was no significant difference between the mean titre of male and female cats using a Mann-Whitney test (*p* = 0.2). (**B**) Bar chart showing the total % seropositivity for samples from male and female cats. The darker coloured bars show neutralising seropositivity and the lighter stacked bars show non-neutralising seropositivity. On a Fisher’s Exact test, the difference in total seroprevalence between male and female cats was found to be statistically significant (*p* = 0.0089) (depicted with an **), however, there was no significant difference between the neutralising seroprevalence of male and female cats (*p* = 0.8) (depicted as ns).

Male and female cats showed a similar strength of antibody response against SARS-CoV-2; no significant difference was found between the mean neutralisation titres of samples from male and female cats (*p* = 0.24) (Figure 5A). There was also no significant difference found between the neutralising seroprevalence in male and female cats (*p* = 0.87). However, based on the ELISA results, male cats were found to have a significantly higher SARS-CoV-2 seroprevalence than females (*p* = 0.0089) (Figure 5B).

**Figure 6:**
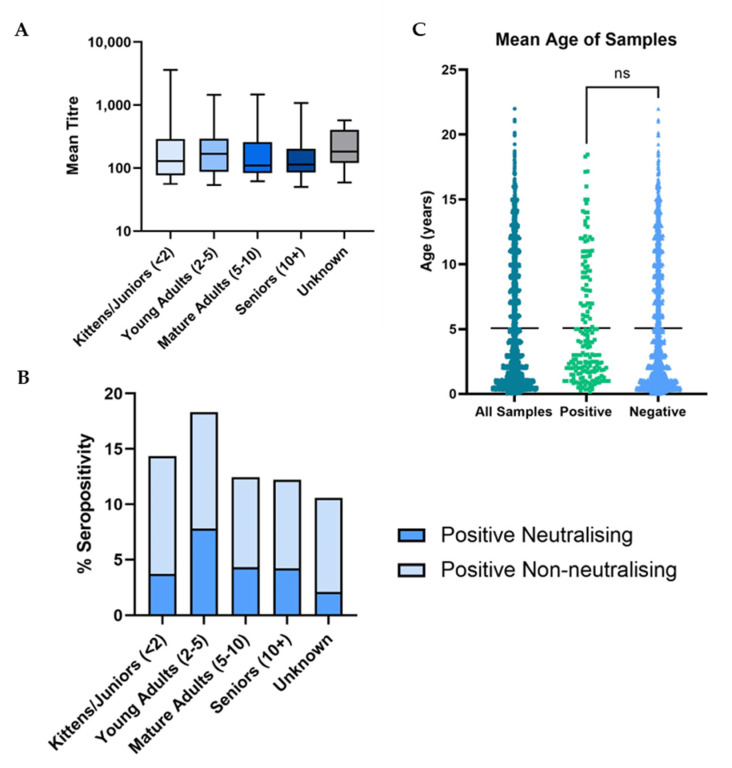
(**A**) Boxplot showing the mean neutralising titre of samples from cats in different age groups. (**B**) Bar graph showing total % seropositivity in different age groups. The darker bars show neutralising seroprevalence and the lighter stacked bars show non-neutralising seroprevalence. (**C**) Scatterplot showing the mean age of sampled cats. Mean age is shown as a black line. There was found to be no significant difference in mean age of cats with positive and negative results (*p* = 0.12).

There was no significant difference found between the age of cats providing positive and negative samples (*p* = 0.12), with a mean age of approximately five years for both groups (Figure 6C).

It was found that cats in the young adult age category were significantly more likely to be seropositive (*p* = 0.0024), as well as neutralisation seropositive (*p* = 0.0002) than those in the other age groups (Figure 6B). Despite this result, the strength of the immune response, in terms of antibody titre, remained consistent across all age groups (Figure 6A) with no significant difference between the mean neutralisation titres of the cats across all five groups (*p* = 0.75).

As the majority of postcode areas had a small sample size, no analysis of seroprevalence was conducted on individual postcode areas. When organised by country, however, there was found to be no significant difference in seroprevalence between samples originating in Scotland, England or Wales (*p* = 0.13). Due to the small sample size, samples from Northern Ireland and the Channel Islands and the Isle of Man were not included in this analysis.

## 4. Discussion

The seroprevalence of SARS-CoV-2 neutralising antibodies in cats of 4.4% described here represents an increase on the figure of 3.2% for February 2022 reported previously [14]. Combining the seroprevalence measured by the assay for SARS-CoV-2 neutralising antibodies and the RBD ELISA suggested a total seroprevalence of 13.9%, indicating that the majority of antibodies detected by the RBD ELISA (68.3%) are non-neutralising. Accordingly, estimates of seroprevalence based on neutralisation assays alone may underestimate the true seroprevalence. As samples with SARS-CoV-2 neutralising activity had a higher mean ELISA OD than non-neutralising samples, the higher seropositivity measured by RBD ELISA may suggest that the neutralising antibody response wanes more rapidly than the overall anti-RBD response and that the excess of non-neutralising samples detected with the RBD ELISA may consist of samples from animals in which either (i) no neutralising antibodies were elicited or (ii) the neutralising response has waned. The non-neutralising component of the anti-RBD response may contribute to protective immunity by processes such as opsonisation as documented in human cases of SARS-CoV-2 infection [45]. Additionally, feline SARS-CoV-2 infections may elicit an antibody response to other viral proteins, such as the nucleocapsid which is known to elicit a strong but transient immune response in humans [46].

It can be observed from serology that the pattern of infections in cats throughout the pandemic appears to mirror the waves of human infections, particularly for the B.1, Alpha and Delta variants. For Omicron BA.1 and subsequent variants, however, the serological picture appears to be more complex; this is likely due to accumulating levels of immunity against older variants combined with recent infections or re-infections with the less immunogenic Omicron variants. It is therefore possible that low Omicron spike immunogenicity in cats has resulted in a decline in detectable seroprevalence despite there being no decline in the actual number of feline infections. Further testing of immunity to more conserved regions on viral proteins that are less antigenically variable than the spike, for example, the nucleocapsid [47], may provide further clarity in this regard. In contrast to humans, SARS-CoV-2 seroprevalence in cats is unaffected by vaccination and so the true picture of the immune response generated against these novel variants in the population may be observed.

It has been previously documented that the Omicron BA.1 variant and its sub-variants are less immunogenic in humans than the ancestral B.1 variant [36,48,49]. During the Omicron wave in early 2022, there was significant concern about this variant due to its high transmissibility [50], its propensity for immune escape [36,51] and vaccine-based immune evasion [36,52,53]. These adaptations are believed to have arisen from alterations in the cell entry mechanism of the virus combined with changes in the antigenicity of the Omicron spike protein [36,54]. In companion animals, Omicron has been reported to be less virulent than previous variants [55], and cats are also believed to be less susceptible to infection with Omicron compared to pre-Omicron variants [56]. These combined factors may account for the low number of Omicron-dominant samples observed in this study.

This study found certain demographic groups of cats had a higher SARS-CoV-2 seroprevalence than others. Purebred cats appeared more susceptible to the virus than non-purebred cats. This may be due to purebred cats being more likely to be kept indoors [57], leading to increased proximity and interactions with their human owners and consequently, an increased risk of infection. Additionally, different pedigree breeds have genetic propensities for different behavioural patterns and “personalities” [58] so this difference in seroprevalence may be due to the cat’s lifestyle. It may be hypothesised that there may also be a genetic component to this higher susceptibility in pure-bred cats. Due to the strict breeding of purebred cats, a number of breeds are known to be more susceptible to certain diseases or abnormalities [59,60] and it is possible that immune abnormalities in certain pedigree breeds could contribute to this increased susceptibility to SARS-CoV-2.

Cats between the ages of two and five had higher seroprevalence than other age groups. This could not be attributed to a stronger SARS-CoV-2 neutralising antibody response, however, as mean neutralisation titres were similar across age groups. It is possible that this higher seroprevalence was due to particular behavioural patterns in this group of cats resulting from hormonal differences.

Male cats were found to have a higher seroprevalence than females. Again, this is likely due to a confounding variable such as behaviour. It has been documented that male cats tend to be “friendlier” to their owners than female cats and have increased contact with them [61]. This higher male SARS-CoV-2 seroprevalence could be a reflection of this greater level of close contact with their human owners. It is also a possibility that male cats are inherently more susceptible to SARS-CoV-2—males have been found to have a higher susceptibility to a number of other viral diseases including feline infectious peritonitis [62] and FIV [63,64].

It should be noted that the samples analysed in this study were collected as residual material from routine diagnostic testing, and thus the majority of samples were collected from cats that were under observation at the time of sampling, whether for a suspected illness, pre-breeding testing or a routine follow-up for an existing condition. A third, 33%, of this dataset, comprised purebred cats, whereas purebred cats are believed to make up just 10% of the UK’s feline population [65,66]. This overrepresentation of samples from purebred cats may have biased the estimated seroprevalence.

In the absence of sequence data with which viral lineages may be assigned, it was not possible to accurately determine whether the dominant variant inferred by serology was the same variant that actually infected the cat. Further studies that incorporate viral genomic sequencing and PCR testing of suspected cases are merited and would determine more accurately the infecting variant as well as detect onward transmission events between cats, humans and potentially other species. It is known that dogs are also susceptible to SARS-CoV-2 [24,67,68] and as they are in frequent contact with both humans and cats, there is a requirement for broader studies to investigate comprehensively within-household transmission dynamics. Similarly, further research into the contribution of the non-neutralising antibodies detected by RBD ELISA to the broader humoral immune response could shed more light on the nature of protective immunity to SARS-CoV-2 in cats.

## 5. Conclusions

The relatively stable seroprevalence of SARS-CoV-2 in cats despite the lower immunogenicity of the Omicron variant further impresses the need for more widespread testing. With an estimated 11 million pet cats in the UK [69], a seroprevalence of 13.9% equates to ~1.5 million SARS-CoV-2 infected animals. Availability of testing and further information on the virulence and pathogenicity of SARS-CoV-2 in companion animals will assist veterinarians in determining in which cases SARS-CoV-2 infection should be considered a differential diagnosis. Unfortunately, to date, due to the UK Government’s narrow case definition [70], the difficulty of random sampling and the lack of veterinary diagnostic testing for SARS-CoV-2, research efforts to deduce the clinical signs and long-term effects associated with feline infection with this virus have been somewhat hampered. Only by utilising broad research methodology can we clearly understand the role of cats in the transmission of SARS-CoV-2, as well as ascertain the true impact of the virus on both animal health and the continuing threat of infection to humans and other species.

## Figures and Tables

**Table 1:** Table showing the SARS-CoV-2 neutralising antibody titres against different variants for the seven post-Omicron dominant samples in the dataset. The dominant titre for each sample is highlighted in green. ELISA OD is shaded in shades of blue according to strength with higher OD shown as a darker shade.

Sample	Sampling Date	ELISA OD	B.1 Titre	Alpha Titre	Delta Titre	BA.1 Titre	BA.2 Titre	BA.5 Titre	BQ.1.1 Titre	XBB Titre
A	20/05/2022	0.94035	0	0	0	79	309	0		
B	31/08/2022	0.5318	0	0	0	0	58	121		
J	09/09/2022	0.5393	0	0	0	0	0	80		
M	14/11/2022	3.0546	0	0	0	69	328	1320		
X	09/01/2023	0.9723	0	74	0	59	124	448	267	0
T	16/01/2023	0.5637	0	0	0	55	342	393	533	68
D	22/02/2023	0.2621	0	0	0	0	61	332	391	0

## Data Availability

The data presented in this study are available in Supplementary Materials.

## Supplementary Materials

The following supporting information can be downloaded at: https://www.mdpi.com/article/10.3390/v15081661/s1.
