# Supplementary material for: Increase in SARS-CoV-2 Seroprevalence in UK Domestic Felids Despite Weak Immunogenicity of Post-Omicron Variants
Source: Viruses. 2023 Jul 30;15(8):1661. doi: 10.3390/v15081661 (PMC10458763; doi:10.3390/v15081661)
Supplement: Supplementary file 1 [file viruses-15-01661-s001.zip › supplementary/supplementary text.docx]

***Section 1. Mutations in Pseudotype Constructs***

Constructs bore the following mutations relative to the Wuhan-Hu-1 sequence (GenBank: MN908947):

- **B.1 (Wuhan-Hu-1 D614G)** – D614G
- **B.1.1.7 (Alpha)** – Δ69-70, Δ144, N501Y, A570D, D614G, P681H, T716I, S982A, D1118H
- **B.1.617.2 (Delta)** – T19R, G142D, Δ156-157, R158G, L452R, T478K, D614G, P681R, D950N
- **B.1.1.529 (Omicron BA.1) -** A67V, Δ69-70, T95I, G142D/Δ143-145, Δ211/L212I, ins214EPE, G339D, S371L, S373P, S375F, K417N, N440K, G446S, S477N, T478K, E484A, Q493R, G496S, Q498R, N501Y, Y505H, T547K, D614G, H655Y, N679K, P681H, N764K, D796Y, N856K, Q954H, N969K, L981F
- **Omicron BA.2 -** T19I, △24/26, G142D, V213G, G339D, S371F, S373P, S375F, T376A, D405N, R408S, K417N, N440K, S477N, T478K, E484A, Q493R, Q498R, N501Y, Y505H, D614G, H655Y, N679K, P681H, N764K, D796Y, Q954H, N969K
- **Omicron BA.5** - T19I, △24/26, △69/70, G142D, V213G, G339D, S371F, S373P, S375F, T376A, D405N, R408S, K417N, N440K, L452R, S477N, T478K, E484A, F486V, Q498R, N501Y, Y505H, D614G, H655Y, N679K, P681H, N764K, D796Y, Q954H, N969K
- **BQ.1.1** - T19I, △24/26, △69/70, G142D, V213G, G339D, R346T, S371F, S373P, S375F, T376A, D405N, R408S, K417N, N440K, K444T, L452R, N460K, S477N, T478K, E484A, F486V, Q498R, N501Y, Y505H, D614G, H655Y, N679K, P681H, N764K, D796Y, Q954H, N969K
- **XBB -** T19I, △24/26, V83A, G142D, △144, H146Q, Q183E, V213E, G339H, R346T, L368I, S371F, S373P, S375F, T376A, D405N, R408S, K417N, N440K, V445P, G446S, N460K, S477N, T478K, E484A, F486S, F490S, Q498R, N501Y, Y505H, D614G, H655Y, N679K, P681H, N764K, D796Y, Q954H, N969K

***Section 2. Analysis of novel ELISA protocol***

In the absence of an acknowledged gold standard test for the detection of anti-SARS-CoV-2 antibodies in cats, the ELISA developed in this study was tested against two established protocols - the Pseudotype-based virus neutralisation assay detailed in section 2.3 of the main text, and the Microimmune SARS-CoV-2 Double Antigen Bridging Assay Kit (COVT016) (Clin-Tech, Guildford, England).

Two different positive OD cut-off values were selected – one stringent (≥0.5 -considered the positive cut-off), and one less stringent (≥0.1 and <0.5 – considered the equivocal cut-off).

Sensitivity and specificity comparisons of this ELISA are detailed below:

|  | 0.1 cut-off (DABA comparison) | 0.5 cut-off (DABA comparison) | 0.1 cut-off (Pseudotype neutralisation comparison) | 0.5 cut-off (pseudotype neutralisation comparison |
| --- | --- | --- | --- | --- |
| Sensitivity | 95.54% | 83.93% | 99.45% | 79.07% |
| Specificity | 30.19% | 75.47% | 35.17% | 89.15% |
| PPV | 74.31% | 87.85% | 6.77% | 25.63% |
| NPV | 76.19% | 68.97% | 99.93% | 98.90% |

It should be noted that neither of these methods are completely suited to an accurate comparison with the ELISA. The Pseudotype Neutralisation assay, while accurate, is only able to detect virus neutralising antibodies which directly prevent cell entry. This accounts for the apparent low specificity of the ELISA, as the ELISA would be predicted to detect any feline anti-RBD antibodies, both neutralising and non-neutralising. At the OD 0.1 cut-off, the ELISA detected 99.45% of all neutralisation positive samples and additionally had a negative predictive value of 99.93%, demonstrating it has a high capacity to detect such antibodies when present.

When comparing this ELISA to the commercial DABA kit, it should be noted that only 165 samples were tested using the DABA compared to over 4000 tested with the neutralisation assay. Additionally, very few neutralisation negative samples were tested with the DABA. Generally, only neutralisation positive samples were tested for confirmation of positivity, as production of the kit had ceased, therefore samples could not be screened in large quantities. Both of these conditions could have affected the results of this analysis.
